# Distance effects in electrochemical micromachining

**DOI:** 10.1038/srep31778

**Published:** 2016-09-01

**Authors:** Lizhong Xu, Yue Pan, Chuanjun Zhao

**Affiliations:** 1 School of Mechanical Engineering, Yanshan University, Qinhuangdao, 066004, China

## Abstract

Considering exponential dependence of currents on double-layer voltage and the feedback effect of the electrolyte resistance, a distance effect in electrochemical micromachining is found, namely that both time constant and double-layer voltage depend on the separation of electrodes. The double-layer voltage is the real voltage used in processing. Under DC voltage, the apparent voltages between two electrodes are constant for different separations, but the real voltages change with the separations. Small separations exert substantial effects on the real voltages. Accordingly, a DC-voltage small-separation electrochemical micromachining technique was proposed. The double-layer voltage drops sharply as the small separation increases. Thus, the electrochemical reactions are confined to electrode regions in very close proximity even under DC voltage. The machining precision can be significantly enhanced by reducing the voltage and separation between electrodes. With this technique, the machining of conducting materials with submicrometre precision was achieved.

The machining of materials at micrometre and nanometre scales is considered a cutting-edge science and technology research field. Electrochemical micromachining has demonstrated excellent development potential in micromachining materials because the approach can transfer materials at the ion scale (metal ions are less than 10% the size of a nanometre)^1^,^2^,^3^. Conventional electrochemical machining has a machining resolution shortcoming that limits micromachining ability. Numerous studies have investigated means of improving machining resolution^4^,^5^,^6^,^7^,^8^,^9^. Among the proposed methods, using ultra-short voltage pulses was a crucial breakthrough. This technique uses ultra-short voltage pulses with nanosecond durations, thereby substantially increasing the machining resolution in the electrochemical micromachining^9^. Using this method, the time constant for charging the double layers on the electrodes is small enough for considerable charging only when electrode separation is in the nanometre-to-micrometre range. This technique marked remarkable progress in electrochemical micromachining and drew worldwide attention. Subsequent research has suggested means of further increasing the micromachining precision of the technique, such as by improving the shape of the electrode^10^,^11^, reducing the duration of the ultra-short pulse^12^, and optimising the duty cycle of the pulse^13^. Using the ultra-short voltage pulse technique has enabled the electrochemical micromachining of NiTi shape memory alloys^14^, corrosion-resistant nickel-based superalloys, Hastelloy B-2^15^, microgrooves and gears on stainless steel plates^16^,^17^, and complex nanostructures including 3D structures, curved features, arrays, and lines in nickel^18^. Various simulation approaches have been proposed for species concentration, temperature distribution, and evolution during pulse electrochemical machining^19^,^20^,^21^,^22^. Computational models have also been developed for simulating the electrochemical process, including the charging and discharging of electrochemical double layers, electric field variation in electrolytes, and feature profile evolution during electrochemical etching^23^,^24^,^25^,^26^. Hybrid tooling with ultra-short voltage pulse electrochemical machining was used to add microstructures to premilled moulds and meso/micro/nanoscale ultra-precision machining was achieved.

The aforementioned studies have all included the following four phases: (1) the development of processing technology, (2) the machining of new materials and structures, (3) the creation of a computational model and simulations of electrochemical micromachining, and (4) hybrid tooling, including with the electrochemical micromachining technology.

However, in the literature, the entire voltage between two electrodes is applied to the double layers, causing an inaccurate prediction of the voltage across the double layers. Controlling the machining accuracy of the ultra-short pulse technique is undesirable because the double-layer voltage is the real voltage used in processing. Because of the exponential dependence of the current on double-layer voltage and the feedback effect of electrolyte resistance, potential drops across electrolyte resistance and potential drops in double layers were investigated in this study. The voltages across the double layers and those between two electrodes were distinguished clearly. The distance effects of the potential drop across double layers in electrochemical micromachining were found to include the distance effects of time constant and voltage amplitude. This research fills the gap left by previous studies, which have only investigated the time constant distance effect.

Our findings can be used as follows: (1) to accurately predict the time constant of the transient response of the double-layer voltage and properly determine pulse duration in the ultra-short pulse electrochemical micromachining, and (2) to propose a novel electrochemical micromachining technique based on the voltage amplitude distance effect. In this study, a DC-voltage small-separation electrochemical micromachining technique was proposed and used for the local etching of stainless steel and nickel film. By using the technique, electrochemical machining with submicrometre precision was achieved.

## Results

### Distance effects of the voltage drop across the double layers

**Figure 1:**
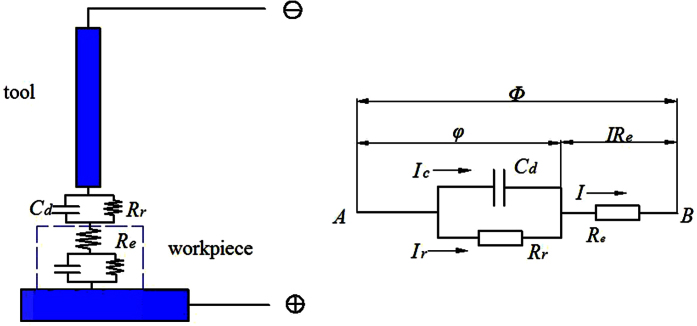
Equivalent circuit of two electrodes immersed in electrolyte. φ is the voltage in the double layers, R_e_ is the electrolyte resistance, R_e_ = dρ_L_, ρ_L_ is the electrolyte resistivity, d is the separation between two electrodes, Φ is half of the voltage between two electrodes, I_c_ is the charging current in capacitance, I_c_ = C_d_dφ/dt, C_d_ is the capacity of the double layers, t is time, I_r_ is the current of the electrochemical reaction, I_r_ = 2i_0_shβ(φ − φ_e_), i_0_ is the exchange current density, β = αnF/RT, the transfer coefficient α = 0.5, φ_e_ is the equilibrium potential, φ − φ_e_ is the overpotential.

An equivalent circuit of two electrodes immersed in electrolyte is shown in Fig. 1^9^. From it, we can obtain its equivalent circuit equation R_e_C_d_dφ/dt + φ + 2R_e_i_0_shβ(φ − φ_e_) = Φ (see method section). In existing methods, the electrolyte resistance R_e_ is considered to be quite small and the term 2R_e_i_0_shβ(φ − φ_e_) is not considered. Thus, the entire voltage between two electrodes is considered to be applied to the double layers, i.e. φ = Φ(1 − exp(−t/τ)); here, τ = R_e_C_d_ = dρ_L_ C_dL_, where C_dL_ is the capacity of the double layers per unit area.

**Figure 2:**
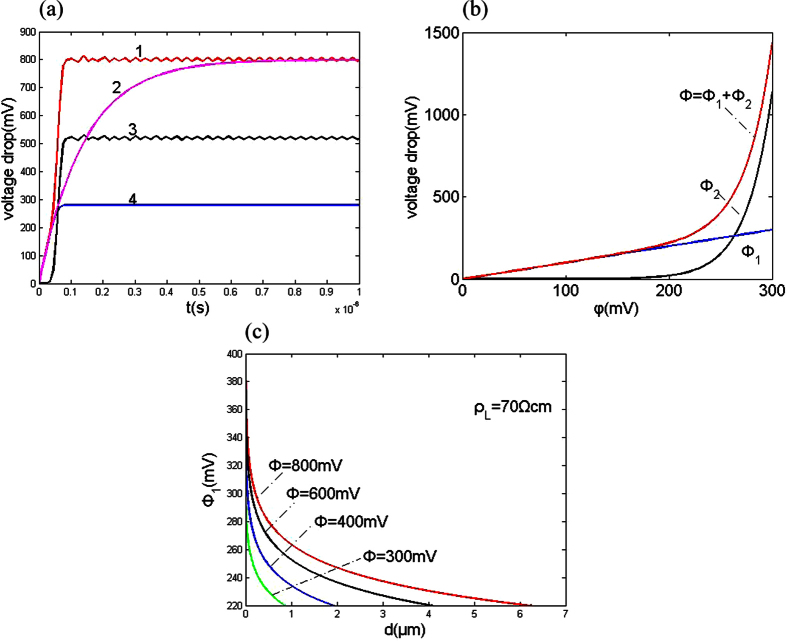
Simulation results. (**a**) Step voltage responses between two electrodes. line 1, half of the voltage drop between two electrodes; line 3, the voltage drop across electrolyte resistance *R*_e_; line 4, the voltage drop across the double layers; line 2, the voltage drop across the double layers from equation *φ* = *Φ*(1 − exp(−t/τ)); *d* = 2 μm, *i*_0_ = 1 mA/cm^2^, ρ_L_ = 70 Ωcm, C_dL_ = 10 μF/cm^2^, Φ = 800 mV. (**b**) Voltages as a function of the voltage drop across the double layers. blue line, the voltage drop across the double layers; black line, the voltage drop across resistance *R*_e_; red line, half of the total voltage between two electrodes. (**c**). Changes of the voltage across the double layers along with separation *d* for different *Φ*.

Using our circuit equation and the same parameters as a previous study^9^, we investigated step voltage responses between two electrodes (Fig. 2a). Here, as the resting potential of the surface is adjusted to the Cu/Cu^2+^ equilibrium potential, the voltage φ in the double layers directly corresponds to the overpotential (φ − φ_e_) for Cu dissolution. Figure 2a shows that for the aforementioned parameters, the voltage drop across the electrolyte resistance R_e_ is larger than that across the double layers and thus should not be neglected; the voltage drop across the double layers is much smaller and its time constant is much shorter than those indicated using existing equations. The circuit nonlinearity causes the time constant to decrease and the voltage responses to fluctuate. Figure 2b gives the voltages as a function of the voltage drop across the double layers (here, Φ = Φ_1_ + Φ_2_, Φ_1_ = φ, and Φ_2_ = 2 R_e_i_0_shβ(φ − φ_e_)). Figure 2b shows that for the aforementioned parameters, the voltage drop across the double layers is nearly equal to the total voltage for φ smaller than 200 mV; because φ is above 200 mV, the voltage drop across resistance R_e_ grows quickly and equals φ at 250 mV. Above 250 mV, the voltage drop across resistance R_e_ becomes much larger than that across the double layers. This result shows that the equation φ = Φ(1 − exp(−t/τ)) can be used only for a small voltage drop across the double layers because it would cause a large error for a relatively large voltage drop across the double layers.

When the separation *d* is decreased to a submicrometre scale, the voltage drop across the double layers is clearly increased. However, it remains much smaller than half of the total voltage between two electrodes. This is because of the strong negative feedback effect of the electrolyte resistance caused by the exponential dependence of the reaction current on the overpotential. Thus, the term 2 Re i_0_shβ(φ − φ_e_) in the equivalent circuit equation should not be neglected, even if the separation *d* is decreased to a submicrometre scale. Figure 2c depicts the voltage across the double layers as a function of the separation *d* and voltage Φ. The figure reveals that the voltage across the double layers can be increased substantially by reducing the separation *d* when the voltage Φ between two electrodes is small.

The aforementioned research has three main findings. (1) The obtained time constant is much shorter than that determined using existing equations. (2) The voltage drop across the double layers is much smaller than that determined using existing equations. (3) Both the time constant and voltage drop across the double layers depend on the separation between the electrodes. As the separation increases, the time constant grows and the voltage drop across the double layers decreases. We call the third finding the distance effects of the voltage drop across the double layers; specifically, this refers to the distance effect of the time effect constant and the distance effect of the voltage amplitude. Previous studies have examined only the time constant distance effect.

### Static-voltage small-separation electrochemical micromachining technique

Our findings can be used for the following. (1) To accurately predict the time constant of the transient response of the voltage across the double layers and to properly determine pulse duration t_on_ to increase the machining resolution of the current ultra-short-pulse electrochemical micromachining. The time constant obtained using existing methods is a few times more than our calculated value. On the basis of our method, the spatial resolution (effective distance *d*) as a function of the pulse duration was calculated for ultra-short-pulse electrochemical micromachining. Our results were consistent with the experimental results of Schuster (see method section). (2) To propose novel electrochemical micromachining techniques based on the findings of the voltage amplitude distance effect. This effect also occurs under static voltage conditions if an electrochemical reaction occurs. Under a pulse voltage, the voltage amplitude distance effect of the transient response is identical to that under a static voltage. The voltage across the double layers is the real voltage for processing, and half of the voltage between two electrodes is the apparent voltage. The real voltage depends on the processing accuracy and speed. Under a static voltage, the apparent voltage is constant for the different separations between the electrodes, but the real voltages differ substantially from each other for the different separations. During a small separation, the effects of the separation between the electrodes on the voltage amplitude across the double layers become considerable. On the basis of the voltage amplitude distance effect of the double layers under a small separation, a novel electrochemical micromachining technique can be proposed: static-voltage small-separation electrochemical micromachining.

Using this technique, a small static voltage between two electrodes is used. As soon as a small separation between the electrodes is achieved, a relatively large voltage across the double layers can be obtained (Fig. 2c). Here, a separation of approximately 2 μm corresponds to Φ_1_ = 250 mV for Φ = 800 mV, and a separation of approximately 0.5 μm corresponds to Φ_1_ = 250 mV for Φ = 400 mV. For Φ = 400 mV, the voltage across the double layers decreases sharply as soon as the separation between the electrodes is larger than 0.5 μm. Therefore, the electrochemical processes are also sharply confined to electrode regions in close proximity to the tool even though the static voltage is used (here, the apparent voltage is constant 400 mV for the different separations between the electrodes, but the real voltages are quite different for the different separations).

**Figure 3:**
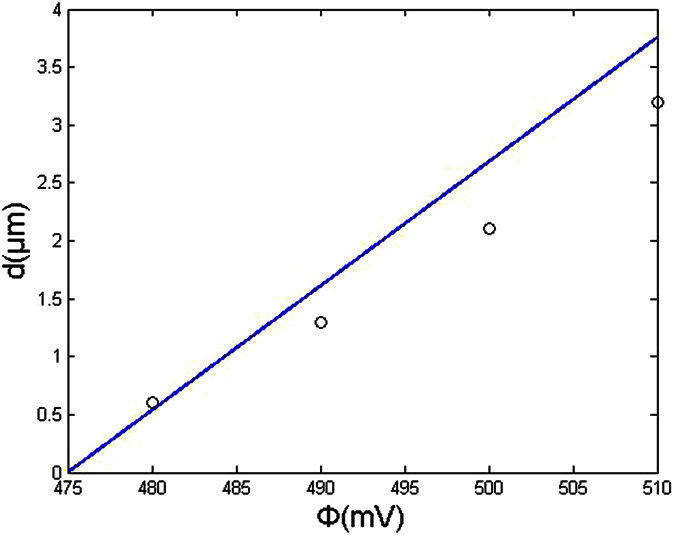
Achievable machining precision as a function of the half of the voltage between two electrodes (electrolyte is 0.1 M H_2_SO_4_). Open circles, test values; solid lines, calculated values.

To obtain the dependence of the achievable machining precision on the voltage between two electrodes, etching experiments on a 5-μm-thick nickel film were performed. The electrolyte was 0.1 M H_2_SO_4_ (see supplementary information A). In this experiment, a cylindrical W wire 10 μm in diameter was used as the tool. Approximately 500 s was required to etch through the nickel film. Afterward, the etching was conducted continuously for approximately 300 s. The experiment was repeated with varying voltages between the electrodes. Figure 3 plots the experimental spatial resolution ((it is equal to the inter-electrode gap and is defined by d = (D_1_ − D_2_)/2, here D_1_ is the width of a machined groove or diameter of a machined hole; D_2_ is the diameter of the tool pole; D_1_ and D_2_ are determined with a microscope.) for different voltages between two electrodes.

In the electrochemical machining Ni film, the electrochemical reaction is the dissolution of Ni at an anode and the generation of hydrogen gas at a cathode. The achievable machining precision can also be calculated from our equation d = (Φ − Φ_1_)/2ρ_L_ i_0_shβ(Φ_1_ − φ_e_). Here, Φ_1_ is considered a Ni dissolution voltage that is obtained using the machining test. Both machining precision *d* and the difference (Φ − Φ_1_) decreases with decreasing Φ (half of the voltage between two electrodes). When *d* reaches zero, the difference (Φ − Φ_1_) decreases to zero and Φ≈Φ_1_. Thus, the Ni dissolution voltage Φ_1_ can be considered a voltage value corresponding to the intersection point of the voltage axis and fit line of the test date in Fig. 3. Thus, we can obtain Φ_1_ (equal to 475 mV). For 0.1 M H_2_SO_4_, the electrolyte resistance is 50 ohm.cm and i_0_ = 1.7 mA/cm^2^. The overpotential (Φ_1_ − φ_e_) equals 175 mV (see supplementary information B).

On the basis of the aforementioned results, the achievable machining precision as a function of the half of the voltage between two electrodes for a specific electrolyte concentration (0.1 M) was simulated (Fig. 3). The results showed that the spatial resolution increases linearly as the voltage between two electrodes was decreased. The experimental results agreed closely with those obtained using the simulation. This indicated that machining accuracy can be significantly enhanced by reducing the voltage between two electrodes.

The initial separation between the tool pole and workpiece has not effect on the machining precision. In electrochemical micromachining, the separation could reach a balance separation automatically (here, the moving velocity of the tool pole is equal to the velocity of the workpiece dissolution). The balance separation is used to show the machining precision. Figure 3 shows that the balance separation decreases and the machining precision is improved when the voltage between two electrodes reduces.

**Figure 4:**
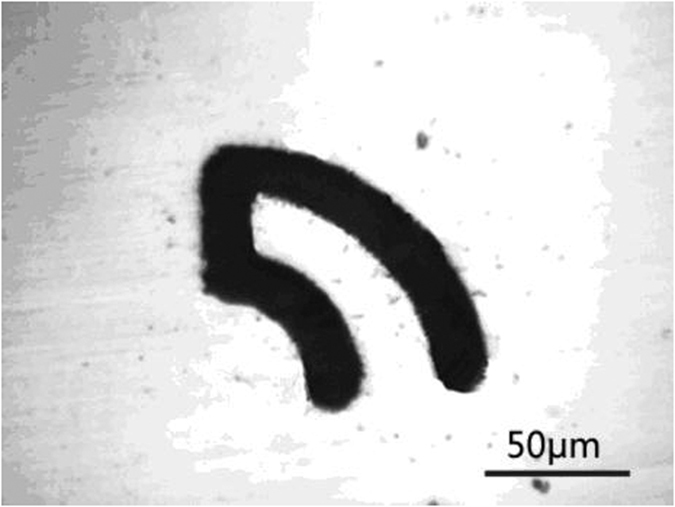
Micro circular cantilever machined with our method.

Using this technique, a microcircular cantilever was machined on a 5-μm-thick nickel film (Fig. 4). Here, a W wire 10 μm in diameter was used as the tool and a solution of 0.1 M H_2_SO_4_ was used. The applied voltage between two electrodes was 1000 mV for etching (half of the voltage between two electrodes was 500 mV). The tool was first etched vertically 7 μm deep, through the workpiece and then the tool was moved laterally along a given path in the nickel film. The tool feed rate was 0.05 μm/s. Regarding the given path, the tool first moved counter-clockwise in a smaller 1/4 circle (r = 35 μm), then moved a section of a straight line (40 μm) before finally moving clockwise in a larger 1/4 circle (r = 70 μm). The average arc length of the machined circular microcantilever was approximately 82 μm, and its width and thickness were 20 and 5 μm, respectively. The width of the trough was 14 μm. The effective distance between the tool and the wall of the trough was approximately 2 μm. The machining precision was nearly one (2 μm); this was achieved using 50-ns ultra-short voltage pulse electrochemical micromachining, as proposed in a previous study^9^. The well-defined shape of the microcircular cantilever demonstrated the high precision of the microstructures achievable with our technique.

In order to finish static voltage-small separation electrochemical micromachining, an intelligent control technique was developed. Here, a threshold current is set at which the tool-workpiece separation is equal to a quite small value. If the loop current is smaller than the threshold current, the tool is driven to feed continuously. As soon as the loop current is equal to the threshold current, the tool stops, which makes the workpiece be machined rapidly under a relatively large current. As the machining causes increase of the tool-workpiece separation, the loop current drops rapidly to one value smaller than threshold current, and then the continuous feed begins again. The threshold current can be determined with test (Supplementary information C). Of course, another larger threshold current is set at which short circuit occurs. As soon as the loop current gets to the threshold current, the tool goes back 1 μm rapidly.

**Figure 5:**
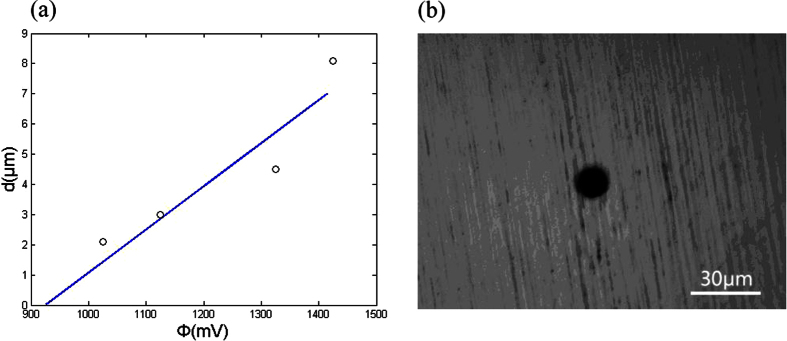
Experimental and simulated results for 0.1 M NaNO_3_. (**a**) Achievable machining precision of a stainless steel film as a function of the half of the voltage between two electrodes; open circles, test values; solid lines, calculated values. (**b**) a micro hole machined with our method.

The proposed technique was used to conduct etching experiments on other electrochemically active materials, using a different electrolyte. Figure 5a shows achievable machining precision of the hole machined on a 5-μm-thick stainless steel film as a function of a half of the voltage between two electrodes. Here, the solution 0.1 M NaNO_3_ was used. A W wire 10 μm in diameter was used as the tool. Figure 5a plots the experimental spatial resolution for different voltages between two electrodes. From the intersection point of the voltage axis and fit line of the test date in Fig. 5a, we can obtain the dissolution voltage Φ_1_ of passive film Fe_2_O_3_ (i.e., 925 mV). At an anode, transpassive reaction occurs from Fe_2_O_3_ to Fe^2+^. Its equilibrium potential is considered to be its transpassive potential (+810 mV). The experimental electrolyte resistance was 18 ohm.cm and *i*_0_ = 0.16 mA/cm^2^. The overpotential (Φ_1_ − φ_e_) equalled 310 mV (Supplementary information B). Thus, using equation d = (Φ − Φ_1_)/2ρ_L_ i_0_shβ(Φ_1_ − φ_e_), the achievable machining precision as a function of Φ can also be calculated. The calculated values are compared with the measurements and favourable agreement is obtained. Figure 5b shows a hole machined in a 5-μm-thick stainless steel film in a solution of 0.1 M NaNO_3_. Here, the tool diameter was 10.2 μm and the hole diameter was 11.7 μm. The machining precision reached 750 nm.

The results show that for different electrochemically active materials, using proper electrolyte solution, submicrometre precision machining can be achieved using our proposed DC-voltage small-separation electrochemical micromachining technique. This method disproves the assumption that only limited spatial resolution of approximately 0.1 mm can be achieved when using a DC voltage^9^. This study also illustrates the existence of a distance effect of the voltage drop across the double layers in electrochemical micromachining.

## Discussion

Compared to work of previous workers, our research has three main differences: (1) find distance effects of the potential drop across double layers in electrochemical micromachining which includes time constant distance effect and voltage amplitude distance effect. In previous studies, only time constant distance effect is noticed. (2) From the distance effects, accurately predict time constant of the transient response of the double-layer voltage in the ultra-short pulse electrochemical micromachining. The time constant from previous methods is a few times more than our calculated value. (3) propose a novel electrochemical micromachining technique based on the voltage amplitude distance effect: a DC –voltage small-separation electrochemical micromachining technique. It brakes through the traditional concept of only achieving limited spatial resolution of about 0.1mm with the DC voltage^9^. The most important advantage of our technology is that only a DC power supply is needed (without needing expensive ultra-short pulse supply). With a DC power supply, only spatial resolution of about 0.1mm can be achieved from traditional concept. With our technology, the sub-micrometer machining precision was achieved with a DC power supply.

## Methods

### Equivalent circuit equation

For the equivalent circuit of two electrodes immersed in electrolyte, using Kirchhoff’s voltage law, we know


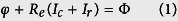


where *φ* is the voltage in the double layers, *R*_e_ is the electrolyte resistance, *R*_e_ = *d*ρ_L_, ρ_L_ is the electrolyte resistivity, *d* is the separation between two electrodes, Φ is half of the voltage between two electrodes, *I*_c_ is the charging current in capacitance, 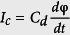, *C*_*d*_ is the capacity in the double layers, *t* is time, *I*_*r*_ is the current of the electrochemical reaction, 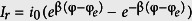, 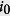 is the exchange current density, 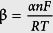, α is the transfer coefficient, *φ*_*e*_ is the equilibrium potential.

Substituting *I*_c_ and *I*_r_ into Eq. (1), yields


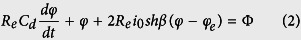


Equation (2) is just the equivalent circuit equation of two electrodes immersed in electrolyte.

### Analytical solutions

Equation (2) can be resolved by the numerical method. In order to control machining accuracy of the ultra-short pulse electrochemical micromachining easily, an approximate analytical solution of Eq. (2) can be given as well. Letting φ = Φ_1_ + Δφ and substituting it into Eq. (2), neglecting high order terms, yields


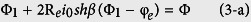



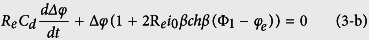


From Eq. (3-a), the voltage drop Φ_1_ across the double layers and the voltage drop Φ_2_ across resistance Re after transient response can be determined. From Eq. (3-b), the transient response of the voltage across the double layers can be given (here, −Φ_1_ is the initial value of Δφ)


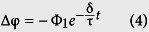


where τ = dρ_L_ C_dL_ and 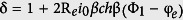


Thus


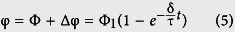


Results show that the analytical solution is in good agreement with numerical solution when parameter δ is taken to be two times of the average value from zero to Φ_1_, i.e.


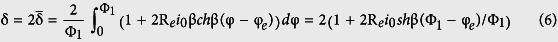


**Figure 6:**
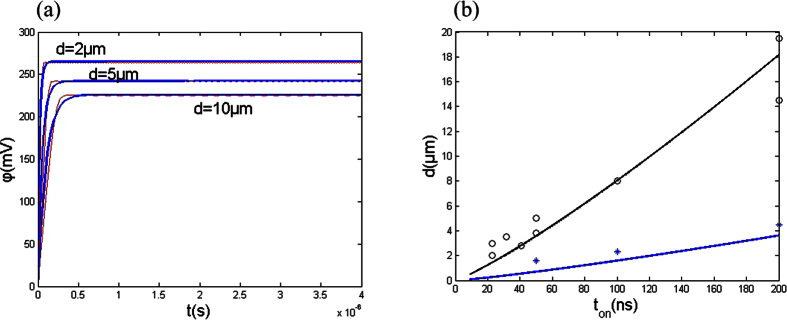
(**a**) Comparison between analytical and numerical solutions for several different separation *d*. blue line, analytical solution; red line, numerical solution. (**b**) Comparison between calculated and experimental results of the spatial resolution; open circles, test values for electrolyte resistance of 30 Ωcm; asterisk, test values for electrolyte resistance of 150 Ωcm.

Using Eqs (2)–(6), 14, 8, 9, 11, 12, the comparison between the analytical and numerical solutions is done (see Fig. 6a). Figure 6a shows that the analytical solution is in agreement with numerical solution for several different separations.

The equivalent time constant of the transient response of the voltage across the double layers is 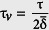 and the condition for effective charging double layers is (here, t_on_ is pulse duration)


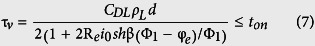


Thus


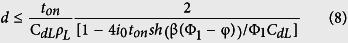


From Eq. (3a), Φ_1_ can be determined by the distance *d*. Then, substituting the distance *d* and Φ_1_ into Eq. (7), the equivalent time constant τ_ν_ can be calculated. Thus, the dependence of the spatial resolution (effective distance *d*) on the critical pulse duration can be obtained (see Fig. 6b). Figure 6b gives calculative spatial resolution (effective distance *d*) as a function of the pulse duration (here Φ = 800 mV). It shows that the analytical results using Eq. (7) is consistent with the experimental results from Rolf Schuster^9^.

## Additional Information

**How to cite this article**: Xu, L. *et al.* Distance effects in electrochemical micromachining. *Sci. Rep.* **6**, 31778; doi: 10.1038/srep31778 (2016).

## Supplementary Material

Supplementary Information
