# Supplementary material for: Distance effects in electrochemical micromachining
Source: Sci Rep. 2016 Sep 1;6:31778. doi: 10.1038/srep31778 (PMC5007593; doi:10.1038/srep31778)
Supplement: Supplementary Information [file srep31778-s1.pdf]

Distance effects in electrochemical micromachining

Lizhong. Xu[1]*, Yue. Pan[1,+], Chuanjun. Zhao[1,+]

[1]School of Mechanical Engineering, Yanshan University, Qinhuangdao, 066004, China

*Corresponding. xlz@ysu.edu.cn.

[+]these authors contributed equally to this work.

Supplementary information

A. Optimum of the electrolyte concentration

Effects of the electrolyte concentrations on the achievable machining precision were investigated and the optimum electrolyte concentration was determined. In etching experiments on a nickel film 5μm thick, several different H_2SO_4 concentrations (0.05M, 0.1M, 0.15M and 0.20M) were used. The experimental spatial resolution is plotted for different electrolyte concentrations (see Figure S1). Here, half of the voltage between two electrodes is 500mV. Figure S1 shows that the spatial resolution decreases gradually, gets to a minimum value at 0.1M, and then increases rapidly with H_2SO_4 concentrations. So, H_2SO_4 electrolyte concentration is taken to be 0.1M when machining micro circular cantilever on a nickel film.

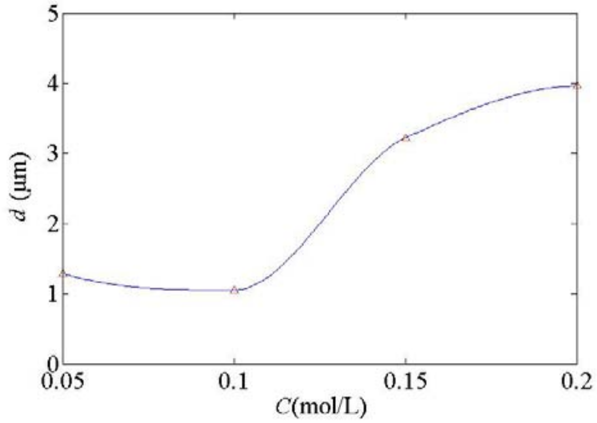

Figure S1. Effects of the electrolyte concentrations on the achievable machining precision; triangles, test values; solid lines, fited values.

B. Determination of the over-potential

In the electrochemical machining Ni film, the electrochemical reaction is the dissolution of Ni at an anode and the generation of hydrogen gas at a cathode[30]. At the anode, a passive film forms on the surface and the reaction current is very low. As the potential increases, the passive film is broken, and the reaction current increases, which is called trans-passive dissolution. Here, the workpiece potential should be located in the region between the passive region and the trans-passive region. Therefore, the equilibrium potential at the anode should be taken as its passive potential near to trans-passive region which can be obtained by test (it is 600mV). At the cathode, the electrochemical reaction is: $2H^{+}+2e=H_2$. In 0.1 M H_2SO_4, the electrolyte resistance is 50ohm.cm and i_0=1.7mA/cm^2 (ref. 31), and PH value is small and the equilibrium potential is near to zero (it is taken as 0mV). Thus, the electromotive potential caused by the equilibrium potentials at anode and cathode is 600mV-0mV=600mV.

As Φ_1 is equal to 475mV, the total over-potential at anode and cathode is equal to 2x475mV-600mV=350mV. In the electrochemical machining, a balance electrode is used to control the over-potential between the workpiece and electrolyte to be equal to half of the total over-potential. So, in equation $d=(\Phi-\Phi_1)/2\rho_L\ i_0 sh\beta(\Phi_1-\varphi_e)$, $\Phi_1-\varphi_e$ is equal to 175mV.

In solution of $NaNO_3$, stainless steel is in passive state and Fe_2O_3 is formed on the stainless steel. So, at an anode, trans-passive reaction occurs from Fe_2O_3 to Fe^{2+}. Its equilibrium potential is taken as its trans-passive potential (+810mV[32]). At a cathode, the electrochemical reaction is still the generation of hydrogen gas. Here, PH value is 7. So, the equilibrium potential for reaction $2H^{+}+2e=H_2$ is -420mV. Thus, the electromotive potential caused by the equilibrium potentials at anode and cathode is 810mV+420mV=1230mV.

From intersection point of the voltage axis and fit line of the test date in Fig.5A, we can obtain dissolution voltage Φ_1 of Fe_2O_3 (it is 925mV). The experimental electrolyte resistance is 18 ohm.cm. In order to fit a simulated data to the experimental data, i_0 is was determined to 0.16mA/cm^2.

The total over-potential at anode and cathode is equal to 2x925mV-1230mV=620mV. In the electrochemical machining, a balance electrode is also used to control the over-potential between the workpiece and electrolyte to be equal to half of the total over-potential. So, in equation $d=(\Phi-\Phi_1)/2\rho_L i_0 sh\beta(\Phi_1-\varphi_e)$, $\Phi_1-\varphi_e$ is equal to 310mV.

C. Determining threshold current

In order to finish static voltage-small separation electrochemical micromachining, an intelligent control technique was developed. Here, the current-feedback control is used. As the tool is near to the workpiece, the current flowing through the cell grows significantly. The current value directly monitors the tool-workpiece separation, which is used in a feedback loop for automated adjustment of the gap width between tool-workpiece.

A threshold current is set at which the tool-workpiece separation is equal to a quite small value. If the loop current is smaller than the threshold current, the tool is driven to feed continuously. As soon as the loop current is equal to the threshold current, the tool stops, which makes the workpiece be machined rapidly under a relatively large current. As the machining causes increase of the tool-workpiece separation, the loop current drops rapidly to one value smaller than threshold current, and then the continuous feed begins again. Thus, a relatively large current micromachining process is achieved under small voltage. Of course, another larger threshold current is set at which short circuit occurs. As soon as the loop current gets to the threshold current, the tool goes back 1μm rapidly.

Before machining nickel film in solution of 0.1 M H_2SO_4, the threshold current above mentioned was determined by test. Here, the applied voltage between two electrodes is 980mV for etching (half of the voltage between two electrodes is 490mV), and the threshold current was taken as 0.1mA, 0.3mA, 0.5mA, and 1.0mA. The achievable machining precision as a function of the threshold current I_t was given in Figure S2. It shows that the best machining precision with this technique is obtained when the threshold current I_t is taken as 0.3mA. It corresponds to the tool-workpiece separation of about 1.3μm.

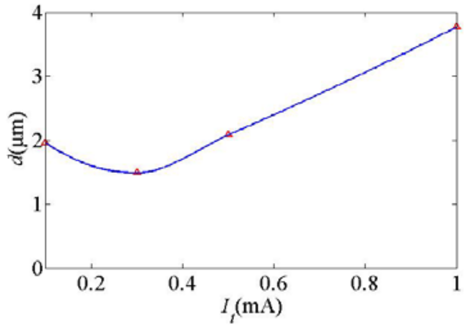

Figure S2. Achievable machining precision as a function of the threshold current I_t (electrolyte is 0.1 M H_2SO_4); triangles, test values; solid lines, fited values.
